# Steerable sheath for exclusively femoral bilateral extension of previous fenestrated endovascular aneurysm repair with iliac branch devices

**DOI:** 10.1016/j.jvscit.2021.04.002

**Published:** 2021-04-24

**Authors:** Roberta Vaccarino, Angelos Karelis, Björn Sonesson, Nuno V. Dias

**Affiliations:** Vascular Center Malmö, Department of Thoracic surgery and vascular diseases, Skåne University Hospital, Malmö, Sweden

**Keywords:** Endovascular aneurysm repair, Branched stent graft, Contralateral approach, Femoral access, Iliac branch device

## Abstract

We report the treatment of type Ib endoleak after fenestrated endovascular aneurysm repair (FEVAR) with iliac branch device (IBD) to allow exclusive transfemoral access without a femoral-to-femoral through-and-through wire. The patient was treated with fenestrated endovascular aneurysm repair and showed expansion of the aneurysm owing to a type Ib endoleak. An IBD was implanted by the use of a contralateral steerable sheath for internal iliac artery catheterizing. A computed tomography scan showed the patency of the target vessels and resolution of the endoleak. The use of a steerable sheath without femoral-to-femoral through-and-through wire to bridge the internal iliac artery in patients receiving an IBD after prior EVAR is feasible and avoids the risks associated with upper extremity access.

Iliac branch device (IBD) implantation after prior endovascular aneurysm repair (EVAR) with a bifurcated stent graft has generally required brachial or axillary access to bridge the internal iliac artery with a covered stent. This approach is associated with risks of arterial and peripheral nerve injury^1^^,^^2^ and intracranial embolic complication owing to catheter manipulations in the aortic arch.^3^ For this reason, anecdotal reports have suggested the use of steerable sheath from the contralateral femoral artery.^4^^,^^5^ However, the stability of the sheath over the flow divider has been poor and a femoral-to-femoral through-and-through wire had to be used. Because this wire needs to be kept in place parallel to the one used for hypogastric access, it limits the available lumen within the steerable sheath and does not allow the complete deployment of the IBD with reestablishment of the flow to the lower limb.

We report the successful use of a contralateral steerable sheath to allow exclusive transfemoral access for IBD implantation without the need for a femoral-to-femoral through-and-through wire. This technique seems to be safe and feasible to avoid upper extremity access.

## Technique

**Fig 1:**
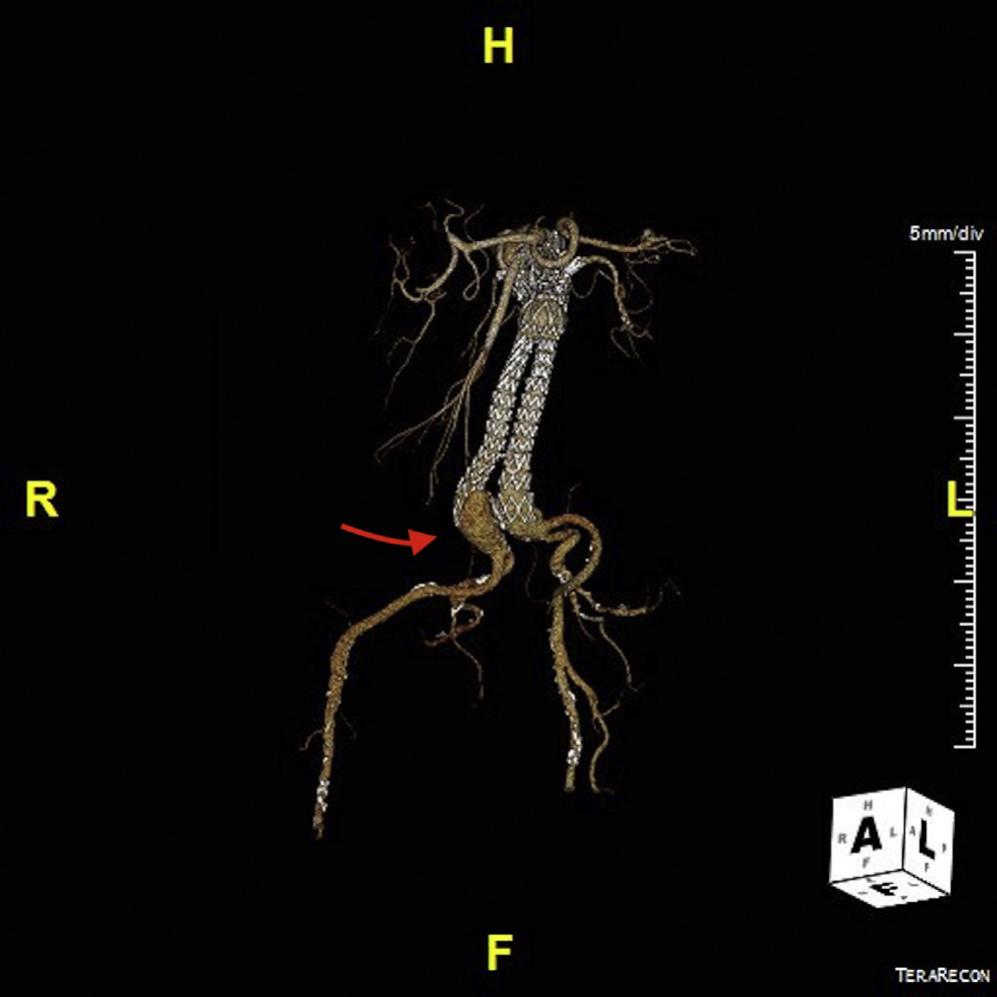
Preoperative three-dimensional reconstruction of the juxtarenal aorta already treated with an infrarenal endovascular aneurysm repair (EVAR) and fenestrated EVAR (FEVAR) extension. The red arrow points to the right-side type Ib endoleak.

A 73-year-old woman presented with asymptomatic bilateral type Ib endoleaks (Fig 1). She had been previously treated with an infrarenal bifurcated EVAR for an asymptomatic 56-mm infrarenal aortic aneurysm. A 26-mm InCraft graft (Cardinal Health, Seoul, Korea) was used inside of the instructions for use (distal diameters of 16 mm and 20 mm). Two years later, a proximal extension with a custom-made modified preloaded triple fenestrated (fenestrated EVAR [FEVAR]) cuff was done (34 mm diameter, 89 mm long; Cook Medical, Bloomington, Ind) because of a type Ia endoleak. Ten days after the FEVAR, the patient was readmitted with an acute type B aortic dissection with FEVAR graft collapse. This condition was treated successfully with thoracic endovascular aortic repair and redilatation of the visceral segment. The computed tomography angiography 1 year after FEVAR showed bilateral type Ib endoleaks and sac expansion to 79 mm.

**Fig 2:**
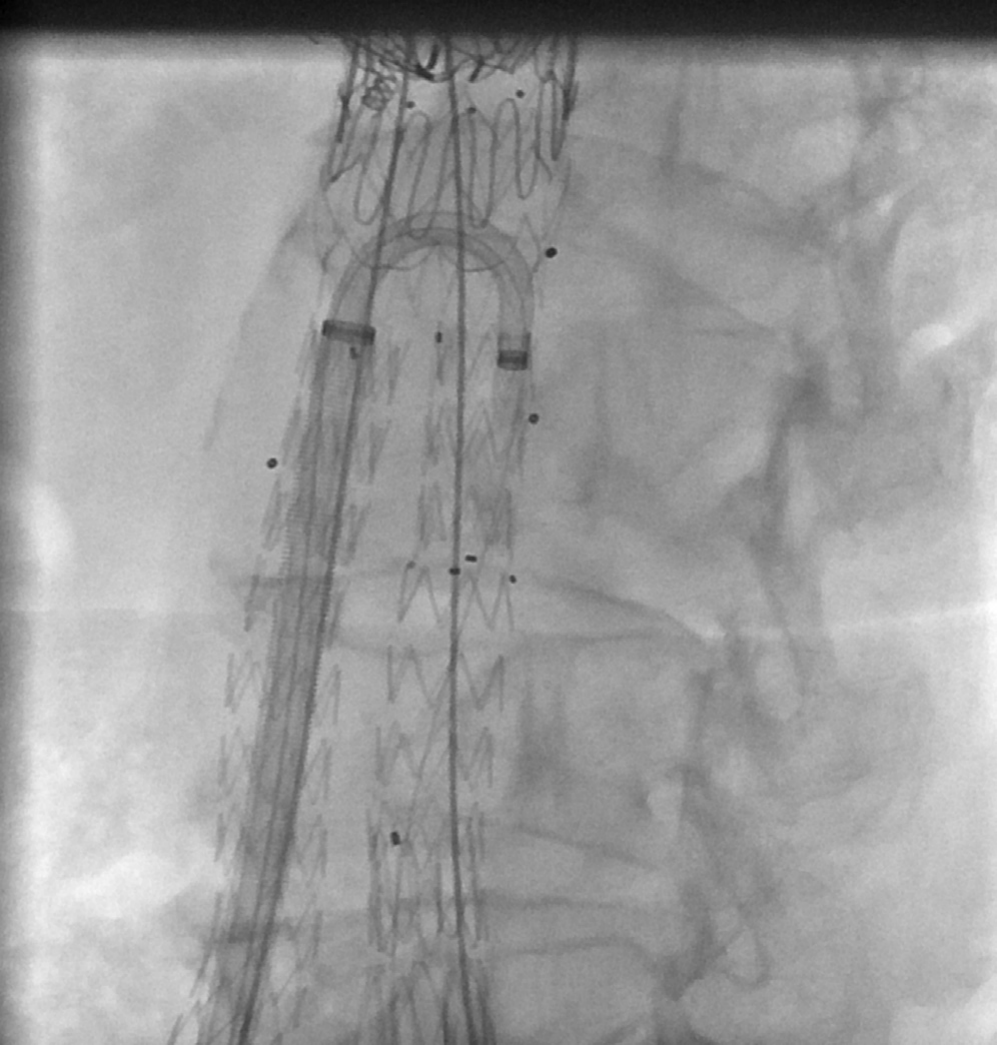
The iliac branched device is completely deployed on the left side. A bidirectional 8.5F, 74 cm long steerable sheath (HeartSpan Medium Curve Angle, Merit Medical, South Jordan, Utah) is introduced from the contralateral transfemoral access into a 12F DrySeal introducer (W. L. Gore & Associates, Inc).

**Fig 3:**
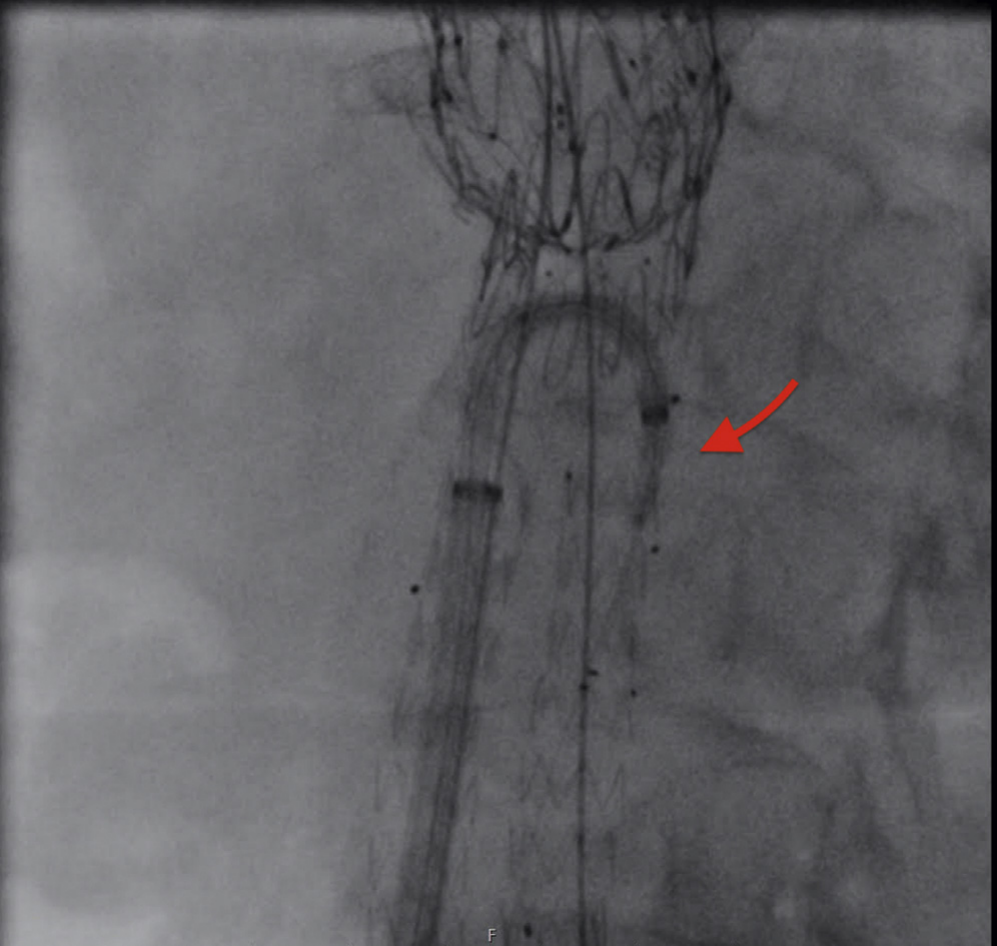
A balloon-expandable covered stent was tracked through the steerable sheath on the right side to bridge the iliac branch device (IBD) into the left internal iliac artery. The *red arrow* points to the stent through the steerable sheath.

Two Proglide devices (Abbott Vascular Inc., Santa Clara, Calif) were placed in each common femoral artery after ultrasound guided puncture under general anesthesia in a hybrid operating theatre (Artis Zee, Siemens, Erlangen, Germany). Intraoperative fusion image guidance with CO_2_ and iodinated contrast was used. A commercially available iliac branched device (ZBIS-10-61-58, Cook Medical) was introduced and deployed completely on the left side without the use of the preloaded catheter. The branch to the internal iliac artery was left temporarily unbridged, a 13-mm self-expanding covered stent was placed proximally (Viabhan; W. L. Gore & Associates, Inc, Flagstaff, Ariz) to connect to the iliac gate of the infrarenal graft and the IBD delivery system was removed using partial traction on the Proglide sutures to downsize the puncture hole. A bidirectional 8.5F, 74 cm long steerable sheath (HeartSpan Small Curve Angle, Merit Medical, South Jordan, Utah) was introduced from the right transfemoral access through a 12F DrySeal Flex sheath (W. L. Gore & Associates, Inc) to minimize manipulation of the Proglide sutures (Fig 2). The internal iliac artery was catheterized and a balloon expandable covered stent (Gore Viabahn Balloon-Expandable Endoprosthesis VBX 8 mm diameter, 59 mm long; W. L. Gore & Associates, Inc) was advanced bare-back and deployed to bridge the IBD into the internal iliac artery (Fig 3). Because the diameter of the most distal common iliac artery was only 15 mm, the proximal part of the external iliac limb of the IBD was reinforced with an uncovered balloon-expandable stent (BeSmooth 10 mm diameter, 38 mm long; Bentley InnoMed GmbH, Hechingen, Germany) with a kissing balloon on the internal iliac artery. Subsequent angiography showed internal iliac artery patency, but there was a small dissection on the distal edge of the bridging stent motivating distal extension with a 10-mm self-expanding stent (Protégé 10 mm diameter, 40 mm long, Medtronic, Minneapolis, Minn). Renewed angiography confirmed the absence of dissection or endoleaks and a self-expanding stent (Protégé 12 mm diameter, 80 mm long; Medtronic) was used to reline the distal external iliac part of the IBD. The steerable sheath was removed and inserted into the other side. The same procedure was then performed on the right side, but without the need for extending the internal iliac artery stent with a self-expanding stent distally. The IBD had the same specifications as on the left side and the external iliac segment was also reinforced and extended with the same type of self- and balloon-expandable stents. The internal iliac artery was bridged with a shorter balloon expandable covered stent (VBX 8 mm diameter, 39 mm long).

Technical success was confirmed by intraoperative angiography and a cone beam computed tomography scan. The total operation time was 210 minutes with the use of 46 mL of iodine contrast (Omnipaque 140 mg I/mL, GE Healthcare, London, UK).

**Fig 4:**
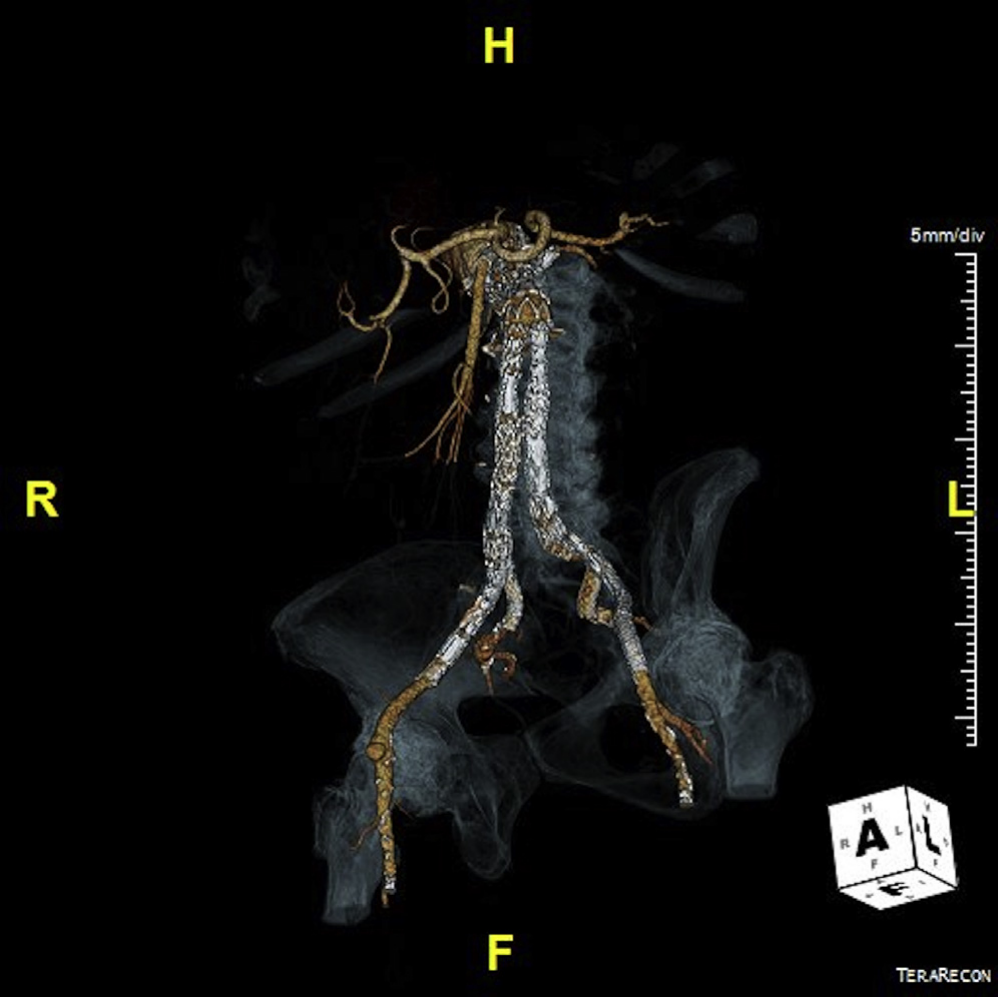
A three-dimensional rendering reconstruction of the aorta at 6 months postoperatively. The image shows patency of both internal iliac artery after the placement of bilateral iliac branch devices (IBDs) with resolution of type Ib endoleaks. The internal iliac artery were bridged with balloon-expandable covered stents.

The patient was discharged after 7 days with no buttock claudication. Six months later, computed tomography angiography confirmed the patency of both internal iliac artery and resolution of the preoperative type Ib endoleaks (Fig 4).

The patient's permission was obtained for the publication of the clinical history, surgical technique, and images.

## Discussion

IBD is an established solution for the preservation of internal iliac artery patency in aneurysms extending to common iliac arteries. Most commonly, bilateral femoral access is used, but when a previous EVAR is in place an upper extremity access is usually required. This situation is associated with a risk of neurologic events,^3^ which increases with an enlarging profile of the sheath.^6^ Moreover, in case of a FEVAR, cranial access is also associated with the risk of damaging the fenestrations during their crossing or tracking of tools through distal tortuous anatomies, which can lead to involuntary movement of the long sheaths. To decrease these risks, other techniques have been used using exclusively femoral accesses. The up-and-over transfemoral technique is generally a suitable alternative.^7^^,^^8^ However, our patient had been previously treated with a FEVAR after a previous infrarenal EVAR, which limits the distance between the lowest fenestrations and the flow divider. For this reason, the up-and-over technique would have resulted in a risk of damaging the side branch stents. Similarly, the use of stabilizing sutures in sheaths, which is otherwise very useful in branch repairs, limits the sheath to engage the branch. In our case, this would mean coming closer to the renal fenestrations with the top of the sheath and potentially endangering them.^9^^,^^10^

Another alternative is the use of a femoral-femoral through-and-through 0.014- or 0.018-inch guidewire to stabilize the sheath. The technique used in the present report avoids this wire since the sheath used provides sufficient stability for access of the internal iliac artery. This not only simplifies the procedure, but also maximizes the inner lumen of the sheath for the bridging stent allowing it to have an inner diameter of only 8.5F. These are two of the important issues to take into consideration when performing branched repairs from a femoral approach. First, there is a need for the sheath not to lose its downwards curvature when a bridging stent is racked through the curvature. Second, the sheath needs to maintain its inner lumen on the curvature to allow the passage of the stents. Moreover, the bridging stents need to have a shaft with flexibility and pushability to cope with the 180° curvature. Even if the materials have become better, the combability balance between sheath and stents needs to be tested until dedicated tools become available.

Importantly, this technique allows the complete deployment of the IBD with immediate downsizing of the access. In this way, perfusion of the lower extremities is not significantly decreased at any time, which may have significance for the prevention of spinal cord ischemia in cases like this one with extensive aortic coverage.^11^ The absence of the small wire also avoids the snaring step, making the procedure more expeditious. Finally, one reservation needs to be made on the advancement of the covered bridging stents without a sheath placed into the internal iliac artery. This was possible with the device used, but combability needs to be checked if other alternatives are chosen.

## Conclusions

The use of a contralateral femoral steerable sheath allows for distal extension of prior FEVAR with IBD only through transfemoral access and without a femoral-to-femoral through-and-through wire. This strategy avoids the risks associated with upper extremity access.
